# Effects of virtual hands and feet on the onset time and duration of illusory body ownership

**DOI:** 10.1038/s41598-022-15835-x

**Published:** 2022-07-12

**Authors:** Ryota Kondo, Maki Sugimoto

**Affiliations:** Department of Information and Computer Science, Keio University, 3-14-1 Hiyoshi, Kohoku-ku, Yokohama, Kanagawa 223-8522 Japan

**Keywords:** Human behaviour, Cognitive neuroscience

## Abstract

In the illusory body ownership, humans feel as if a rubber hand or an avatar in a virtual environment is their own body through visual-tactile synchronization or visual-motor synchronization. Despite the onset time and duration of illusory body ownership has been investigated, it is not clear how the onset time and duration change when a part of the body is missing from the full-body. In this study, we investigated the completeness of the full-body for the illusion onset and duration by comparing the following conditions: complete avatar, avatar missing hands and feet, and avatar with hands and feet only. Our results suggest that avatar hands and feet only shorten the duration of the illusion, and missing body parts, such as only hands and feet or no hands and feet, reduce the sense of body ownership and of agency. However, the effects of avatar completeness on the onset time are unclear, and no conclusions can be made in either direction based on the current findings.

## Introduction

### Illusory body ownership by visual-tactile synchronization

The sense that a body belongs to oneself is called the sense of body ownership^1^, and illusory body ownership is known to occur in bodies other than one’s own. For example, in the rubber hand illusion (RHI), brushing the rubber hand and the participant’s hand simultaneously creates a rubber hand feel as if it were one’s own hand^2,3^. The same procedure can induce the illusion of a full-body. For example, stimulating the participant’s body and mannequin with a brush and observing the brush strokes from the mannequin’s head position through a head-mounted display (HMD) can help induce illusory body ownership of the mannequin^4^. Thus, visual-tactile integration generates an illusion for another body part or the full-body.

### Visual-motor synchronization

Illusory body ownership can also be generated by presenting visual stimuli synchronized with a participant’s movements. For example, in the moving rubber hand illusion^5,6^ and virtual hand illusion^7^, the participant’s hand and the mannequin’s hand, or the virtual hand, moved in synchrony; the participants felt as if they were their own hands. The same procedure can be applied to a full-body avatar. The avatar moves synchronously with the participant’s movements, making the whole body of the avatar feel as if it were the participant’s own body^8^. The difference from visual-tactile integration is that a sense of agency (the feeling that an action is one’s own^1^) is generated. However, multiple studies have shown the sense of agency over movements of an innate body even without visuomotor synchronization and in the absence of participant movements^9–11^.

### Visual-proprioception synchronization

Studies have reported observation of a mannequin or virtual body presented in the same position as a participant’s body generating illusory body ownership^12,13^, suggesting that the congruence of proprioception with the posture and position of the visual body is sufficient to induce illusory body ownership. Several studies have demonstrated the importance of non-innate body posture and distance. RHI does not occur when a rubber hand is rotated by 90°^3,14^ or 180°^5,15^. Furthermore, ownership is weaker when a rubber hand is farther away^16,17^. The full-body illusion was weaker in the third-person perspective than in the first-person perspective^10,18–20^. Moreover, the full-body illusion does not occur when the virtual body is at a distant location^21^.

### Illusion onset time and duration

As discussed above, multisensory integration through temporal and spatial congruence is essential for creating this illusion. Thus, for what time does sensory integration produce this illusion? Furthermore, how long does the illusion persist?

Kalckert and Ehrsson^22^ showed that the RHI was induced in approximately 23 s. Lloyd^17^ manipulated the distance between a rubber hand and a participant's hand, and simultaneously measured the onset time. The average onset time for all participants was approximately 15 s in the fastest condition (distance of 27.5 cm), and the average for only the participants who experienced the illusion was approximately 5 s. In a study investigating the onset time and duration of RHI^23^, the onset times were approximately 100 s (Experiment 1) and 50 s (Experiment 2), and the duration was approximately 80 s during a tactile stimulus of 180 s. Their results included participants who had not experienced the illusion; therefore, the onset of the illusion was slower than that in other studies. In their study, the onset time of disownership was measured to be approximately 120 s. The onset of disownership is significantly slower than that of ownership. It might be more difficult for a participant to detect a disownership experience than the more vivid aspects of the RHI, such as the feeling of the rubber hand as one’s or sensing touch on the rubber hand. Abdulkarim^24^ et al. measured subjective feelings of ownership and proprioceptive drift at timings of 0, 20, 40, 60, 120, and 300 s from the end of a rubber hand and a participant's hand stroke in the RHI. They reported a stronger illusion in the subjective feeling of ownership up to 300 s and proprioceptive drift up to 20 s in the synchronous condition than in the asynchronous condition.

These differences in the onset time and duration of the RHI may be due to differences in the experimental setup and procedures. Kalckert and Ehrsson^22^ measured onset time only for participants in whom RHI occurred; therefore, onset time seems to be particularly fast. The relationship between body appearance and onset has also been investigated, with onset time being slower when the skin color of a rubber hand is different from that of the participant^25^. The authors considered that a mismatch between the body model and the visual body slows sensory integration. The visual body and body model is compared in the initial stage of the illusion generation process in the neurocognitive model by Tsakiris et al.^26^.

In the visual-only and visual-motor synchronization conditions, the full-body illusion occurred 5 s, earlier than the RHI^13^. Conversely, in a study that examined the relationship between the body part that presented visual-tactile stimuli and full-body illusion^27^, the earliest condition produced the illusion at 25 s. Additionally, in a study in which the illusion occurred at 5 s^13^, the onset timing was examined by measuring the body ownership at each fixed time. The study illusion produced in 25 s^27^; it was measured by pressing a button when participants experienced the illusion. In addition, they used a statement that explicitly mentioned: “ownership over a whole body” which is different than the typical statements of “ownership of body or hand,” so they explicitly timed whole-body sensations that speculatively might take a little longer to build up, which may have resulted in onset times differences.

The difference in the onset time between the full-body and body parts seems to be influenced by the congruence between the visual stimuli and proprioception. This is due to the presentation of a full-body avatar^13^ or mannequin^12^ in the same position as the participant’s body using an HMD.

### Invisible body illusion

It has been shown that the sense of body ownership occurs not only for a rubber hand and a full-body but also for an invisible hand^28^ and an invisible full-body^29,30^. In the invisible hand illusion^28^, as in the RHI, a brush was used to simultaneously stroke a space and a participant’s hand. Further, the participant felt their hand in the space and the illusion was induced in 9.3 s. Similarly, an invisible full-body is perceived by stroking a space and a participant’s body with a brush and observing the brush strokes with an HMD^29,30^. Our previous studies^31,32^ have shown that an invisible full-body is perceived between the hands and feet and that illusory body ownership is generated by observing the stimulation of only the hands and feet moving synchronously with a participant’s movement. The illusory body ownership generated by an invisible body is as strong as that of a full-body avatar^31^.

However, in a study that manipulated the transparency of a virtual body^33^, body ownership decreased as transparency increased. Nevertheless, because observing the virtual body was the only method used to induce body ownership, visual information was likely weakened in the high-transparency condition, and integration with proprioception did not work effectively. The results suggest that to induce body ownership in an invisible body requires cues to complement the invisible body (such as visual-tactile stimulation with a brush) or virtual hands and feet moving synchronously with a participant’s movement.

### Aim

Although the onset time and duration for the RHI and that for the full-body illusion and invisible hand are known, it is not clear how the onset time and duration change when a part of the body is missing from the full-body, as in the case of an invisible body with only the limbs^31,32^. In this study, we investigated the completeness of the full-body illusion onset and duration by comparing the following conditions: complete avatar, avatar missing hands and feet, and avatar hands and feet only^31,32^. We also measured the strength of body ownership and sense of agency. In terms of multisensory integration, we expected that the illusion would be generated earliest and persist longest in the complete avatar condition with the most visual information. Body ownership and agency were also considered strongest in the complete avatar condition.

## Results

Only participants who scored more than one on the questionnaire for body ownership were measured for onset time and duration. Therefore, the numbers of participants who measured the onset time and duration were 24, 18, and 12 for the complete avatar, avatar missing hands and feet, and avatar hands and feet only conditions, respectively. The participants were asked to press a button on the right-hand controller as quickly as possible when they felt the avatar was as if their own body and kept pressing the button as long as they felt it to measure the onset time and duration. The time at which the button was pressed for the first time was recorded as the onset time. The duration of each trial was recorded as the total time the button was pressed continuously. The questionnaire results, onset time, and duration were tested for normality using the Shapiro–Wilk test. However, they do not follow a normal distribution. The Wilcoxon signed-rank test was used for the questionnaire and the Wilcoxon rank-sum test was used for the onset time and duration. P-values were corrected using the Bonferroni’s method. The number of participants in each condition that the illusion experienced was compared using Fisher's exact test, and p-values were corrected using Bonferroni’s method.

### Questionnaire

**Figure 1:**
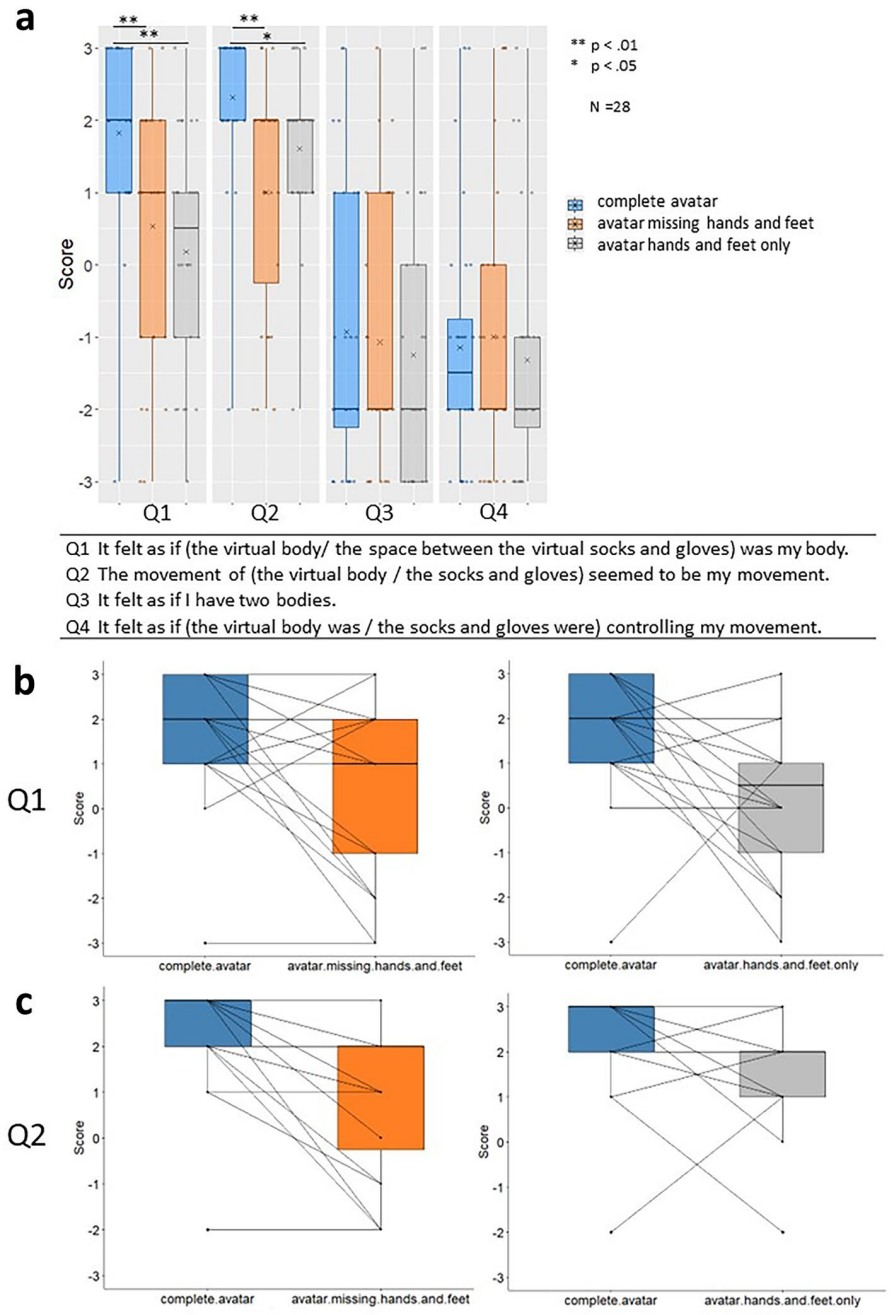
(**a**) All results of the questionnaire. The horizontal thick lines indicate the medians and “x” indicate the mean values. Individual data points are plotted in dots. (**b**) Plots of pairwise comparison lines between individual data points in Q1. (**c**) Plots of pairwise comparison lines between individual data points in Q2.

Body ownership was stronger in the complete avatar condition than in the avatar missing hands and feet and avatar hands and feet only conditions (Fig. 1 Q1: complete avatar vs. avatar missing hands and feet: z = 3.14, p = 0.0029, r = 0.59; complete avatar vs. avatar hands and feet only: z = 3.36, p = 0.0011, r = 0.64). No significant difference was found between the avatar missing hands and feet condition and the avatar hands and feet only condition (Q1: z = 0.74, p = 1.00, r = 0.14). A stronger agency was reported in the complete avatar condition than in the avatar missing hands and feet and avatar hands and feet only conditions (Q2: complete avatar vs. avatar missing hands and feet: z = 3.77, p < 0.001, r = 0.71; complete avatar vs. avatar hands and feet only: z = 2.75, p = 0.013, r = 0.52). There was no significant difference between the avatar missing hands and feet and the avatar hands and feet only (Q2: z = − 1.74, p = 0.26, r = 0.33). Furthermore, no significant differences were found between the control questions (Q3 and Q4).

### Onset time

**Figure 2:**
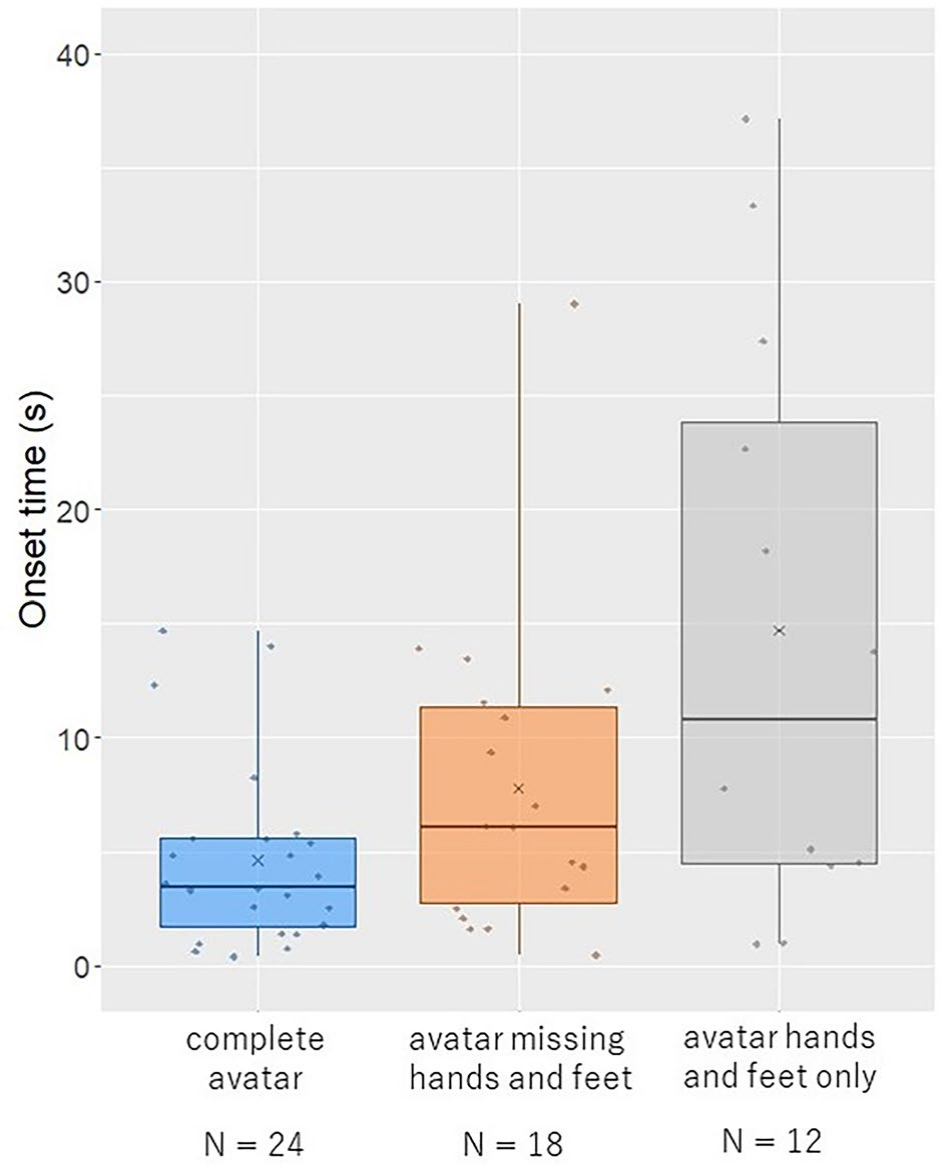
Results of the onset time. The horizontal thick lines indicate the medians and “x” indicate the mean values. Individual data points are plotted in dots.

There were no significant differences between the conditions, although body ownership was induced in the order of complete avatar, avatar missing hands and feet, and avatar hands and feet only (Fig. 2; complete avatar vs. avatar missing hands and feet: z = − 1.6, p = 0.34, r = 0.22; complete avatar vs. avatar hands and feet only: z = − 2.32, p = 0.06, r = 0.32; avatar missing hands and feet vs. avatar hands and feet only: z = − 1.27, p = 0.65, r = 0.17).

### Duration

**Figure 3:**
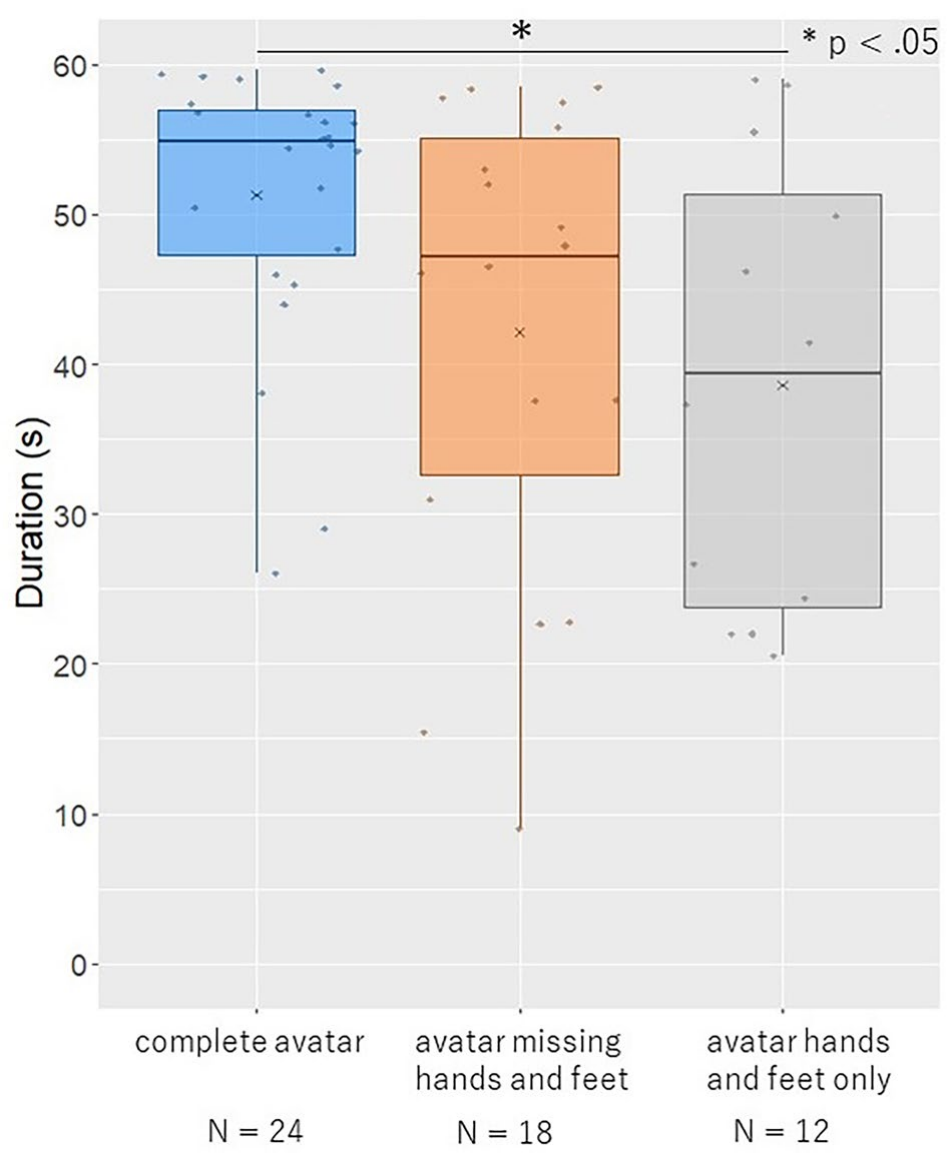
Results of the duration. The horizontal thick lines indicate the medians and “x” indicate the mean values. Individual data points are plotted in dots.

The duration was significantly longer in the complete avatar condition than in the avatar hands- and feet-only condition (Fig. 3, z = 2.38, p = 0.049, r = 0.32). No significant differences were found in the other conditions (complete avatar vs. avatar missing hands and feet: z = 1.93, p = 0.16, r = 0.26; avatar missing hands and feet vs. avatar hands and feet only: z = 0.59, p = 1.00, r = 0.08).

### Frequency of responders vs. non-responders

The results showed significant differences between conditions (p = 0.0039), and multiple comparisons showed significant differences between the complete avatar and the avatar hands and feet only condition (p = 0.0053).

## Discussion

### Summary of results

In this study, we investigated the completeness of the full-body for the illusion onset and duration by manipulating the presenting body area. The results showed no significant difference in the onset time between the conditions. However, body ownership was induced in the order of complete avatar, avatar missing hands and feet, and avatar hands and feet only. The duration was significantly longer in the complete avatar condition than in the avatar hands and feet only condition. In the questionnaire results, body ownership (Q1) and sense of agency (Q2) in the complete avatar condition were significantly more robust than those in the avatar missing hands and feet, and avatar hands and feet only condition. In addition, significantly more participants experienced the illusion in the complete avatar condition than in the avatar hands- and feet-only condition. These results suggest that the avatar hands and feet only shorten the illusion’s duration. Similar to a study in which a part of an arm disappeared^34^, our results also indicate that missing body parts decrease body ownership, agency, and the frequency of responders.

### Illusion generation and persistence process

In the neurocognitive model by Tsakiris et al.^26^, the body model in the brain is compared with the appearance of the fake body in the first stage of the illusion generation process, posture is compared in the second stage, and the body model is updated by multisensory integration in the third stage. Therefore, no body ownership is generated for objects that clearly look different, such as a wooden stick^3^. In addition, body ownership is not generated for fake bodies that are not in an anatomically correct position, such as a rubber hand rotated by 90°^3,14^ or 180°^5,15^. Thus, the influence of body appearance and posture on inducing illusions is significant. In the present study, avatars missing hands and feet or avatar hands and feet only are more likely to be judged as not being a self-body in the first stage of the illusion generation process because it differs significantly from a complete avatar body. The number of participants who experienced the illusion was significantly lower in the avatar hands and feet only condition than in the complete avatar condition.

However, since the onset time and duration were measured only for participants who had already experienced the illusion, it is unlikely that they were judged to be not self-body due to the appearance and posture constraints in the neurocognitive model by Tsakiris et al.^26^. Thus, completeness of the body affects the onset time and duration in the multisensory integration stage. In the present study, there was no significant difference between the conditions in onset time, and the complete avatar condition was significantly longer than the avatar hands and feet only condition in terms of duration. Our results suggest that the completeness of the fake body has an important role in the persistence of illusions. In a study that examined the duration of the RHI induced by 60 s strokes^24^, it persisted even when the eyes were closed once the illusion was induced. This suggests that a body model is formed during the 60 s illusion induction phase, and that visual information is not required for the illusion’s persistence. In the avatar hands and feet only condition, in which the amount of visual information was low, the body model was not updated well by multisensory integration, and the illusion may have been short-lived. Few studies have been conducted on the onset time and duration of changes in body appearance, and further investigation is required.

In the Bayesian causal inference model^35–39^, inferences are performed on whether visual, tactile, and proprioceptive signals are caused by a common source. In the rubber hand illusion^2,3^, tactile and proprioceptive cues from a participant's hand and visual cues from a rubber hand are used to determine whether these multiple sensory inputs originate from the rubber hand or from the participant's hand. In the avatar hands and feet only condition and avatar missing hands and feet condition of this study, body ownership was weaker than in the complete avatar condition because there was less visual information to support that the visual and proprioceptive signals originated from a common body. In the Bayesian causal inference model, the probability that a fake body is one's own gradually increases based on the presented visual and tactile stimuli, and the illusion is induced when the probability exceeds a threshold. In the present study, the illusion was induced earlier as the amount of visual information about the body increased, although no statistically significant difference was found, and our results support this model. In terms of duration, the illusion was weaker and shorter in the avatar hands and feet only condition, probably because the lack of visual cues obscures whether the input signals originated from a common cause.

### Effects of missing body parts

When part of the body was missing, as in the avatar missing hands and feet and avatar hands and feet only conditions, body ownership and sense of agency were lower than those in the complete avatar condition. Additionally, significantly fewer participants experienced the illusion in the avatar hands and feet only condition than in the complete avatar condition. This is consistent with a study that reported a decrease in body ownership and sense of agency when a part of the area between the arm and hand was missing^34^, regardless of the size of the missing area. Conversely, Kilteni et al.^40^ showed that illusory body ownership does not decrease, even when the tip of a virtual arm is missing. In their study, the tip of the arm disappeared after body ownership was induced in the normal virtual arm, suggesting that changes from the body that generated body ownership can be adapted to the body missing a part of the arm.

### Comparison with a study that measured onset time of the full-body illusion

In this study, the onset time of the illusion in the complete avatar condition was 4.6 s, which is similar to a study that measured the onset time of the full-body illusion^13^. These results indicate that the ownership of a full-body avatar is robust and has an extremely fast onset. Conversely, using a mannequin’s full-body illusion requires approximately 25 s^27^. Therefore, visual-motor synchronization may be more likely to produce this illusion. In visual-tactile synchronization, the spatial congruence of the mannequin or virtual body with the physical body is important for the illusion, but in addition, in visual-motor synchronization, correlated movements are probably a crucial extra cue driving the illusion. In terms of the illusion’s strength, one study reported that the illusion is stronger in visual-motor synchronization than in visual-tactile synchronization^41^. In their study, they measured the duration of the illusion by asking participants to verbally report when the illusion was broken and estimated the strength of the illusion from the number of times it was broken using the stochastic model of Slater and Steed^42^. This measurement method could be used in the present study.

### Comparison with our previous invisible body studies

In Kondo et al.^31^, virtual gloves and socks synchronized with the participant's movements were presented 2 m in front of the participant. Consequently, the full-body was perceived in the space between the virtual gloves and socks, and a full-body illusion was generated. The generated full-body illusion was as strong as the full-body avatar presented in front of the participants. In contrast, the full-body elicited more substantial body ownership than limbs alone in the present study. Kondo et al.^31^ presented stimuli in front of a participant, improving the visibility of the entire stimuli and promoting the perception of the invisible body through the completion of the hands and feet. Therefore, it is possible that the completion of the invisible body by the hands and feet did not work well from a first-person perspective, resulting in lower body ownership. Moreover, one minute of learning—less than five minutes of learning in the study by Kondo et al.^31^—might have been insufficient to induce strong ownership of the invisible body.

Our another invisible body study^32^ showed that the spatial relationship of hands and feet is important for the full-body illusion, but the study did not compare it with the full-body avatar. The present results suggest that the spatial relationship between the hands and feet is not sufficient, and completeness of the body is important for the full-body illusion.

### Limitations

This study measured the onset and duration of illusory body ownership by pressing a button. However, we did not measure the strength of the illusion when pressing the button. Therefore, it is unclear whether the illusion was continuously more substantial or whether it occurred discretely. In future studies, we will investigate the body ownership process through continuous measurement. Apart from the missing body parts, it is also possible that the smaller the body area, the weaker the sense of ownership and agency and the shorter the duration. Nevertheless, we did not perform an accurate comparison of body areas. In the future, we will develop stimuli with the same body area but different body parts and compare them to determine whether body area or body part is more critical for inducing and sustaining illusory body ownership.

## Methods

### Participants

Twenty-eight naïve participants (all males, mean age 22.8 years old ± 2.4 SD, mean height 169.7 cm ± 6.7 SD, 26 right-handed, two left-handed) provided written informed consent and participated in the experiment. The participants were recruited from Toyohashi University of Technology. The sample size was calculated using G*Power3.1^43,44^ (repeated measures ANOVA, three avatar conditions, medium effect size f = 0.25, α = 0.05, power (1 − β) = 0.8). The participants had healthy vision and physical motor skills. The Ethical Committee for Human-Subject Research at Toyohashi University of Technology approved the experiment, and all methods were performed in accordance with relevant guidelines and regulations.

### Materials

**Figure 4:**
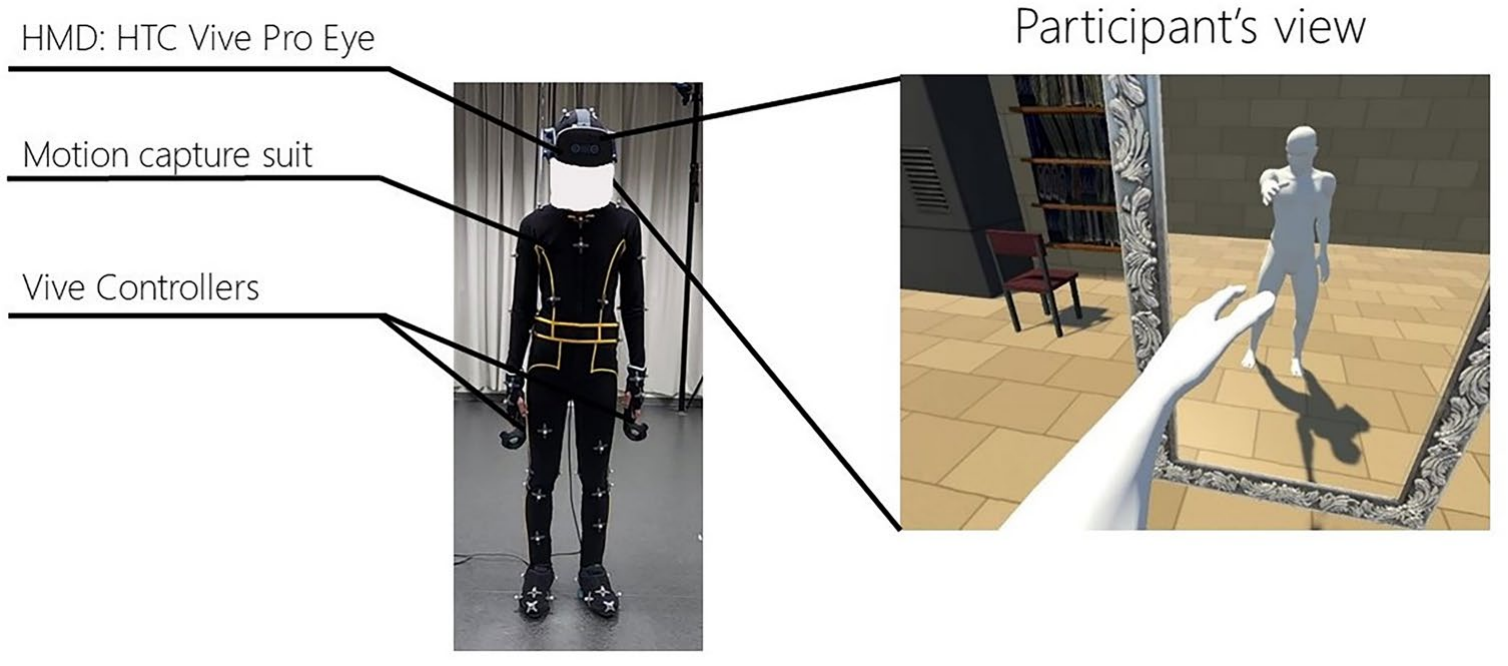
A participant wearing the devices (left) and the participant’s view (right). The figure on the right was made with Unity 2017.4.5f1 (unity.com).

Participants observed the stimuli created by a computer (OS: Windows 10 (64bit), CPU: Intel Core i9-9900 K, RAM:32 GB, GPU: NVIDIA GeForce RTX 2080 SUPER 8G) through an HMD (Fig. 4: right, HTC Vive Pro Eye, resolution:1440 × 1600 pixels per eye, field of view:110°diagonal, refresh rate:90 Hz). Furthermore, the participants wore a motion capture suit, and their movements were captured using 12 cameras (OptiTrack PrimeX 22, resolution: 2048 × 1088, frame rate:360 Hz, latency:2.8 ms). Moreover, the participants held two Vive controllers to measure the onset time and duration of body ownership and answered a questionnaire (Fig. 4, left).

### Stimuli and conditions

**Figure 5:**
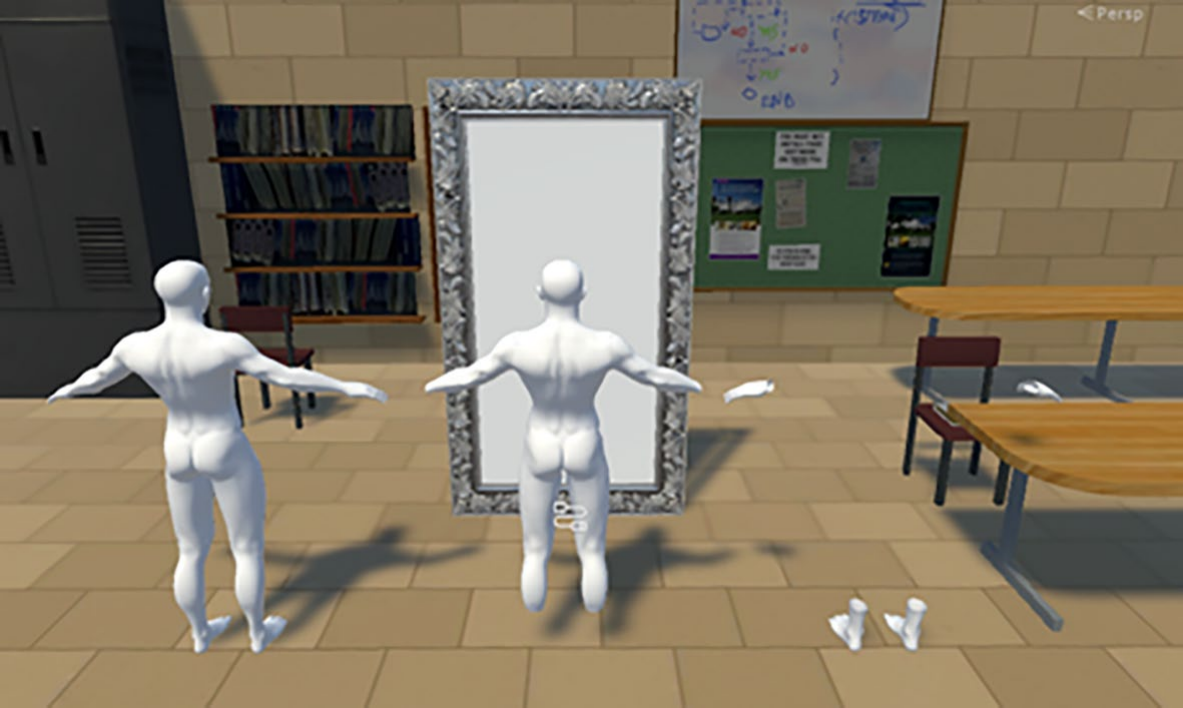
Avatar conditions: complete avatar (left), avatar missing hands and feet (center), avatar hands and feet only (right). The figure was made with Unity 2017.4.5f1 (unity.com).

One of the three types of virtual bodies [Video 1, Fig. 5: complete avatar (left), avatar missing hands and feet (center), and avatar hands and feet only (right)] is presented in the virtual environment. In the complete avatar and avatar with missing hands and feet, a white virtual body was presented to match the avatar hands and feet only condition. The participants observed the virtual body from a first-person perspective and the virtual body moved synchronously with the participant’s movements. In addition, a mirror was placed in front of each participant to reflect the virtual body.

### Procedures

#### Questionnaire session

At the beginning of the experiment, the participants looked around the virtual environment for 30 s to adapt to it. Subsequently, the virtual body was presented and the participants moved following the sound presented at 1 Hz through a pair of headphones for 1 min. The participants were instructed to move their right hand, left hand, right foot, and left foot in that order, and to observe moving body parts during the movement. After the exercise, the participants answered a questionnaire (Table 1) on a seven-point level Likert scale: -3 (did not feel at all), 0 (uncertain), and + 3 (felt very strongly). Three conditions were tested once each in random order.

**Table 1:** Questionnaire items.

Q1	It felt as if (the virtual body/the space between the virtual socks and gloves*) was my body
Q2	The movement of (the virtual body/the socks and gloves*) seemed to be my movement
Q3	It felt as if I have two bodies
Q4	It felt as if (the virtual body was/the socks and gloves were*) controlling my movement

*These statements were used for the avatar hands and feet only condition.

#### Onset time and duration measurement session

For the condition in which the score on the body ownership item of the questionnaire was + 1 or higher, we measured the onset and duration of body ownership. The participants moved for 1 min, as before. Furthermore, when they felt that the virtual body (the space between their hands and feet in the avatar hands and feet only condition) was their body, they were instructed to immediately press a button on their right controller. Furthermore, they continued to press the button as long as they felt it. They were also instructed to press the button when they felt it again, after the illusion disappeared. The time at which the button was pressed for the first time was recorded as the onset time. Furthermore, the total time for which the button was pressed was recorded as the duration of each trial. The onset time and duration were measured three times for each condition in random order.

In both the questionnaire and measurement sessions, the participants removed the HMD after each trial and moved their physical body for approximately 30 s to eliminate the remaining body ownership.

## Supplementary Information

Supplementary Legends.

Supplementary Information.

Supplementary Video 1.

## Data Availability

All data generated or analyzed during this study are included in this published article and Supplementary Data 1.
