# Author Correction: Effects of virtual hands and feet on the onset time and duration of illusory body ownership

**DOI:** 10.1038/s41598-022-19151-2

**Published:** 2022-08-29

**Authors:** Ryota Kondo, Maki Sugimoto

**Affiliations:** Department of Information and Computer Science, Keio University, 3-14-1 Hiyoshi, Kohoku-ku, Yokohama, Kanagawa 223-8522 Japan

Correction to: *Scientific Reports* 10.1038/s41598-022-15835-x, published online 12 July 2022

The original version of this Article contained errors. After publication it transpired that the Supplementary Information file which accompanies the Article, inadvertently contained confidential patient data. This patient data has now been removed from the Supplementary Information file.

Additionally, the legend of Table 1 contained an error, where

“*These statements were used for the limbs-only condition.”

now reads:

“*These statements were used for the avatar hands and feet only condition.”

The original Article has been corrected.

