# Supplementary material for: Effects of virtual hands and feet on the onset time and duration of illusory body ownership
Source: Sci Rep. 2022 Jul 12;12:11802. doi: 10.1038/s41598-022-15835-x (PMC9276807; doi:10.1038/s41598-022-15835-x)
Supplement: Supplementary file 1 — Supplementary Legends. [file 41598_2022_15835_MOESM1_ESM.docx]

**Supplementary Legends**

**Supplementary Video 1**: Avatar conditions: complete avatar (left), avatar missing hands and feet (center), avatar hands and feet only (right)

**Supplementary Data 1**: Questionnaire, onset time, and duration data.
